# Machine learning accurately classifies neural responses to rhythmic speech vs. non-speech from 8-week-old infant EEG

**DOI:** 10.1016/j.bandl.2021.104968

**Published:** 2021-09

**Authors:** Samuel Gibbon, Adam Attaheri, Áine Ní Choisdealbha, Sinead Rocha, Perrine Brusini, Natasha Mead, Panagiotis Boutris, Helen Olawole-Scott, Henna Ahmed, Sheila Flanagan, Kanad Mandke, Mahmoud Keshavarzi, Usha Goswami

**Affiliations:** a Centre for Neuroscience in Education, Department of Psychology, University of Cambridge, UK; b Department of Bioengineering and Centre for Neurotechnology, Imperial College London, UK

**Keywords:** Machine Learning, EEG, Convolutional Neural Network, Developmental Language Disorders, Infancy, Rhythm

## Abstract

Currently there are no reliable means of identifying infants at-risk for later language disorders. Infant neural responses to rhythmic stimuli may offer a solution, as neural tracking of rhythm is atypical in children with developmental language disorders. However, infant brain recordings are noisy. As a first step to developing accurate neural biomarkers, we investigate whether infant brain responses to rhythmic stimuli can be classified reliably using EEG from 95 eight-week-old infants listening to natural stimuli (repeated syllables or drumbeats). Both Convolutional Neural Network (CNN) and Support Vector Machine (SVM) approaches were employed. Applied to one infant at a time, the CNN discriminated syllables from drumbeats with a mean AUC of 0.87, against two levels of noise. The SVM classified with AUC 0.95 and 0.86 respectively, showing reduced performance as noise increased. Our proof-of-concept modelling opens the way to the development of clinical biomarkers for language disorders related to rhythmic entrainment.

## Highlights

• EEG was recorded while 8-week old infants listened to rhythmic speech and non-speech.
• Both A CNN and SVM reliably classified infant brain responses.
• The CNN was more robust to noisy EEG data.
• Simple rhythmic EEG measures may enable prediction of language outcomes.

## 1 Introduction

Developmental speech and language disorders have a relatively high prevalence (~7%, Tomblin et al., 1997) and carry significant risk of life-long difficulties in academic, social, and economic domains (Conti-Ramsden, Durkin, Toseeb, Botting, & Pickles, 2018). Accordingly, the early identification of infants at risk for later language disorders is a priority, as mitigating interventions can be put into place. Yet only half of infants who are “late talkers” will go on to develop language impairment. Late talkers are usually defined as infants aged between 18 and 30 months who, despite having an apparently good understanding of language as well as typically developing motor, cognitive, social, and play skills, have a very limited spoken vocabulary (Moyle, Stokes, & Klee, 2011). Late talkers are typically identified through scoring below a certain threshold on parent-report measures such as the CDI (Fenson et al., 1994) and the ASQ-3 (Squires, Bricker, & Twombly, 2009). Late talkers as a group have poorer language outcomes (Rescorla et al., 1997, Thal et al., 1991). For example, longitudinal data show that a larger proportion of late talkers demonstrate scores below one standard deviation of the mean in spoken language, morphosyntax, and syntax at age 7 (Rice, Taylor, & Zubrick, 2008). It is already recognised that early screening measures could benefit late-talking children, enabling much earlier intervention (Moyle et al., 2011, Roos and Weismer, 2008). Here we report neural data from a longitudinal study based on Temporal Sampling (TS) theory aimed at developing an EEG-based early screening tool suitable for infants, the BABYRHYTHM project (Goswami, 2011). By TS theory, the infant neural response to rhythmic stimuli could provide a potential neural biomarker of risk for later language disorder.

Children with oral developmental language disorder (DLD, previously termed Specific Language Impairment or SLI) and children with developmental dyslexia (a disorder with impaired phonological processing of language) both exhibit impairments in processing rhythm (e.g., Goswami et al., 2011, Cumming et al., 2015). A recent review of child language studies argues for an “atypical rhythm risk hypothesis”, documenting how individuals exhibiting atypical rhythm across both speech-based and non-speech (musical or tapping) tasks are at higher risk for developmental speech/language disorders (Ladányi, Persici, Fiveash, Tillmann, & Gordon, 2020). Despite the growing number of developmental studies showing longitudinal relations between rhythm perception and/or rhythm production and language, *neural* data related to a developmental rhythm hypothesis are sparse. The most comprehensive neural data are currently related to studies testing TS theory, either by using syllable repetition tasks (“ba…ba…”), metronome beats, or filtered speech (Goswami, 2018, for review). TS theory proposes a neural, perceptual, and cognitive framework for understanding atypical phonological development in children. TS theory has also been applied more generally to typical and atypical language development (Goswami, 2019).

The core neural proposal in TS theory is that the automatic alignment of endogenous brain rhythms and amplitude modulation-driven rhythm patterns in speech is atypical for children with developmental dyslexia and DLD (Goswami, 2011). Children with both disorders exhibit impaired amplitude rise time discrimination (Goswami et al., 2002, Corriveau and Goswami, 2009). TS theory proposes that impaired auditory sensitivity to amplitude rise times is related to atypical neural tracking of the speech signal by neuroelectric oscillations (Goswami, 2015). This is because in the adult brain, accurate neural tracking of the speech envelope depends in part on the automatic phase-resetting of brain rhythms by acoustic “edges” (rise times in amplitude associated with syllables) in the speech signal (Giraud and Poeppel, 2012, Gross et al., 2013, Doelling et al., 2014). Amplitude rise times are also important perceptual cues to speech rhythm (Greenberg, 2006). TS theory proposed that neural tracking of *slower rates* of amplitude modulation (<10 Hz, the rates which govern rhythm perception) could be atypical in children with developmental language disorders. Atypical neural tracking and encoding of the speech envelope has indeed been demonstrated for children with dyslexia in both English (Power et al., 2013, Power et al., 2016, Di Liberto et al., 2018) and Spanish (Molinaro, Lizarazu, Lallier, Bourguignon, & Carreiras, 2016). Accordingly, the neural tracking of rhythm by *infants* at risk for developmental language disorders may also be atypical, perhaps from birth or even in the womb, providing a potential biomarker. As a first step towards identifying such biomarkers, here we investigate whether the infant brain responds differentially to speech vs non-speech naturalistic rhythmic stimuli.

Neural prospective studies of language impairment with infants have not to date investigated rhythmic parameters (e.g. Zare, Rezvani, & Benasich, 2016). We thus extended prior neural tracking studies with children to infants. Prior studies testing TS theory have utilised either a rhythmic syllable repetition paradigm or a metronome beat (Power et al., 2012, Power et al., 2013, Colling et al., 2017). Power and colleagues presented children with rhythmic repetition of the syllable “ba” at a 2 Hz rate, in either an auditory-alone (A) condition, an auditory-visual (AV) condition, or a visual-alone (V) condition (a silent talking head). Children with dyslexia showed atypical phase entrainment to speech rhythm in the EEG delta band (not theta band) compared to control children for the AV and A conditions, but not the V condition. The groups were matched for perceptual performance (their accuracy in detecting out-of-time syllables, Power et al., 2013). Colling et al. (2017) recorded EEG while children either tapped to a metronome beat delivered at 2.4 Hz or listened passively. While (following training) tapping synchronization did not differ between children with dyslexia and controls, the dyslexics showed significant neural differences compared to non-dyslexic children in all conditions. Their neural response was out of phase (out of time) with the beat. Accordingly, for child populations, delta-band information may be critical for successful oscillatory entrainment and language development. The focus on delta-band information adopted here is supported by modelling of infant-directed speech (IDS) using an amplitude demodulation approach. This modelling showed that IDS contains significantly greater modulation energy in the delta band (amplitude modulations centred on 2 Hz) relative to adult-directed speech (Leong, Kalashnikova, Burnham, & Goswami, 2017). Linguistic analyses show that stressed syllables are produced on average twice a second across languages (2 Hz, Dauer, 1983). As many languages use prosodic structure (the patterning of stressed and unstressed syllables) as the bedrock of their phonological systems, individual differences in infant neural entrainment to ~ 2 Hz rhythmic inputs may be relevant to developing clinical biomarkers (Goswami, 2019).

We used two naturalistic rhythmic inputs, repetition of the syllable “ta” at 2 Hz (spoken in IDS) and a drumbeat sound, while recording EEG. Although these two rhythmic inputs differ on a range of acoustic parameters, including both spectral and envelope characteristics, they represent two natural inputs with ecological validity matched for rhythmic rate. If a simple drumbeat shows equal sensitivity to speech regarding rhythmic entrainment, this would enable the development of a simple clinical biomarker. We take a machine learning approach to classify syllables from drumbeats in the EEG data. Our goal was binary classification of these rhythmic stimuli, and we utilised two different machine learning approaches.

Many machine learning classifiers have been developed for real world applications. These classifiers can be divided into two main categories: traditional machine learning algorithms and deep learning-based algorithms. Deep learning-based algorithms like CNNs have attracted tremendous interest regarding classification following the introduction of the AlexNet (with more than 75,000 citations) by Krizhevsky et. al in 2012. Among traditional machine learning algorithms, SVMs (Burges, 1998) have achieved state-of-the-art performance on classification tasks. Regarding EEG data, the two approaches have shown mixed results (Garcia et al., 2003, Jrad et al., 2011, Liu et al., 2013, Tang et al., 2017, Lawhern et al., 2018, Lotte et al., 2018). In general, SVMs are efficient in terms of computation and memory, and perform best on small datasets. However, their performance decreases markedly when the data are noisy. CNNs, by comparison, have a high computation cost, require high performance computing units, and their performance can drop significantly due to over-fitting caused by using a small dataset. However, they outperform other methods when the dataset is sizeable (Craik et al., 2019, Roy et al., 2019). As CNNs are more robust against noise, and as infant EEG data are notoriously noisy, here we first build a CNN and then benchmark it against an SVM.

A CNN tasked with classifying labelled groups (e.g., speech vs. non-speech) finds relevant features automatically and iteratively. It works by learning feature representations from the training data, which are then searched for in the test data. CNNs are formed of stacked layers, with each performing a non-linear transformation on its input, and passing the values to the next layer. Initial layers extract low level features, and “deeper” layers extract progressively higher level (more complex) features. To give an intuitive example from computer vision, an initial layer might extract pixel edges, deeper layers would extract shapes based on the edges (horizontal line, circle, triangles), and the deepest layers would classify the shapes (horizon, sun, boat), classifying the overall image as “boat at sea”. By comparison, SVM classifiers are supervised learning algorithms that take manually extracted features as the input and predict the class labels as the output. SVMs learn features extracted from the training dataset and classify the data by finding a hyperplane (whose dimension depends on the number of features), which has the largest possible margins (distances) from different classes, resulting in the highest classification accuracy.

Our core research questions were a) whether a deep learning and machine learning approach could classify the stimulus category from the infant’s brain response, and b) whether our models were robust against an additional level of noise.

## 2 Methods

### 2.1 Participants

EEG was recorded from ninety-five 8-week-old infants (mean age = 62 days, SD = 6.2, 47 female). Infants were recruited as part of a larger study investigating the relation between neural rhythmic entrainment and language acquisition (the BABYRHYTHM project). Primary caregivers provided informed consent in accordance with the Declaration of Helsinki; the study was approved by the Psychology Research Ethics Committee of the University of Cambridge. Study data was stored and managed using the database software REDCap (Research Electronic Data Capture; Harris et al., 2009, Harris et al., 2019).

### 2.2 Stimuli

Infants heard two continuous, non-interspersed, approx. 16-minute streams of either a drumbeat or the syllable /tæ/ repeated at a constant rate of 2 Hz, presented through two speakers. Each full session contained 2000 stimulus repetitions.

### 2.3 EEG recording and pre-processing

Recording took place in an electrically shielded room, adjacent to a control room, primarily in the Cambridge psychology department (Site A). Due to unanticipated building works, 8 (out of 95) infants were recorded instead at a Cambridge hospital site (Site B). This site change enabled us to test whether the CNN was learning regions in high-dimensional space that correlated with noise specific to the recording instrument. The equipment at both sites was from the same manufacturer, EGI. Most parents held their baby near their chest for the duration of the recording session. The room was dimly lit, and infants were encouraged to sleep. EEG data were recorded using a 64 channel EGI Geodesic Sensor Net system at a sampling rate of 1000 Hz. A StimTracker generated an audio mark at the onset of each stimulus, ensuring precisely synchronised events. EEG pre-processing was performed in MATLAB 2018 (MathWorks) using the EEGLAB toolbox (Delorme & Makeig, 2004). The data were first band-pass filtered between 0.5 and 45 Hz. Unused channels 61 to 64 were removed before pre-processing. To reduce computational load, the data were down sampled to 100 Hz. The raw EEG was then assessed for bad channels in a fully automated protocol. If either probability or kurtosis of a single channel was above 2 SD from the mean of all channels, it was interpolated with the average of its closest neighbours, resulting in interpolation of around 20% of channels across all participants (mean = 12.1 channels, SD = 4.6). Channels were then re-referenced to the global average.

To prepare the data for input to the CNN, the continuous EEG data were segmented into two-second non-overlapping epochs, cut to stimulus onset, so that each epoch contained 4 “beats” - either 4 drumbeats or 4 iterations of the syllable. An epoch length of two seconds has been successful in other DL-EEG work (Tjepkema-Cloostermans et al., 2018, van Putten et al., 2018). After segmentation, “noisy” epochs were identified using a function designed to detect improbable data (see MATLAB code). This algorithm marked just over a quarter of epochs for the syllable data (mean 27.26%, SD = 15.82), and a similar number for the drum data (mean 26.83%, SD = 14.37). There are limitations in identifying noisy epochs using a purely automated method and consequently it is rarely the only step taken.

In total, 45,753 two-second drum epochs were recorded, with 34,233 remaining after noisy epoch removal, and 47,604 syllable epochs were recorded, with 33,127 remaining after noisy epoch removal. This yielded 51.9 h of recording for total epochs, and 37.4 h following removal.

### 2.4 Convolutional neural network

The machine learning frameworks Keras (Chollet, 2015) and Tensorflow 1.3 (Abadi et al., 2016), installed on a Dell 7820 machine running Ubuntu 20.04 with a CUDA-enabled NVIDIA Quadro RTX 5000 GPU, were used to build, train, and test a CNN that was inspired by successful architectures presented for DL-EEG work in van Putten et al., 2018, Schirrmeister et al., 2017. The CNN architecture consists of two successive convolutional layers - to perform convolutions over time and then space (Schirrmeister et al., 2017), a max pooling layer followed by dropout, a dense layer, and a sigmoid classification layer (see Table 1). No activation function was used for the first layer; Exponential Linear Units (ELU; Clevert, Unterthiner, & Hochreiter, 2015) were used for all other layers.

**Table 1:** CNN architecture.

Operation	Activation	Filters	Filter size	Dropout rate	Output shape	Parameters
Conv2D		12	1 × 4		60 × 197	60
Conv2D	ELU	12	60 × 4		1 × 194	34,572
MaxPool 2D			1 × 4		1 × 48	
Flatten					576	
Dropout				30%	1 × 48	
Dense	ELU	32			32	18,464
Dense	Sigmoid	1			1	33
**TOTAL**						**53,129**

Craik et al. (2019) showed empirically that using numerical signal values enables a moderate increase in accuracy (from 84% to 87%), therefore we represented the pre-processed EEG as a 2-D array, with channels as rows and time samples as columns. The network predicts two-digit binary numbers [01] or [10] as output, representing either drum or syllable. Data were normalised to a range of [-1, 1] before being presented to the network.

If infants were fussy, the recording session ended early, resulting in fewer data points being collected. To mitigate the resulting class imbalance problem, we apply a class weight function, which generates weights based on the distribution of labels to penalise over or under-represented classes in the training set.

Data from each infant were passed through the network separately. This means that we built 95 distinct networks (one per infant).

After noisy epoch removal, data for each infant consisted of approximately 800 2-D input arrays (with approximately 50% for each class). The *k*-fold (*k* = 5) cross-validation procedure was employed to evaluate and optimize the CNN parameters. As removing noisy epochs resulted in the loss of greater than 25% of data for each stimulus, we subsequently tested the CNN on all the data. To derive the CNN, 80% of data were used as the training data set and the remaining data (20%) as the testing data. The procedure included 4 main stages: 1) shuffle all data randomly; 2) divide data into *k* groups of approximately the same size; 3) for each round: a) let one group be considered as the testing data, (b) let the remaining groups be considered the training data, c) train the model using the training data and test on the testing data, d) calculate the performance score; 4) assess overall model performance using scores obtained for all groups.

**Fig. 1:**
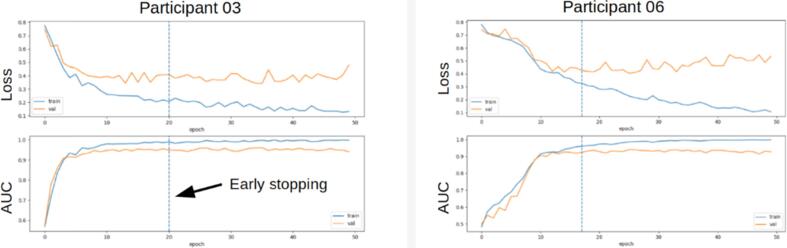
Training and validation plots for two infants from the model development subset for the CNN. Loss and AUC are shown for 50 training runs. Early stopping is marked by the dashed vertical line.

We used systematic trial and error (Chollet, 2018) on a random subset of 8 infants recorded at site A to select the optimum values for the CNN’s hyperparameters, the model development subset. Optimisation was realised using the Adam optimizer (Kingma & Ba, 2014), with learning rate = 0.001, β1 = 0.9, β2 = 0.999, ε = 10^-7^. The loss function was binary cross-entropy, the performance metric was Receiver Operator Characteristic – Area Under Curve (ROC-AUC), and the batch size was 32. We used early stopping, which mitigates overfitting (monitoring the validation AUC with a patience of 6) on the model development subset to determine the optimum number of training runs, yielding a mean of 25 (Fig. 1), thereafter 25 training runs were totally performed.

### 2.5 Support Vector Machine

The algorithm included three steps. 1) *Feature extraction* - in this step, three features including power in delta (1–4 Hz), theta (4–8 Hz), and alpha (8–12 Hz) frequency bands were extracted from each single EEG channel, yielding 180 features for each sample of data. 2) *Feature reduction* - in this step, the number of features were reduced using principal component analysis (PCA), set to yield 30 features. 3) *Classification* - the SVM took the features obtained in the previous step as its input and predicted two-digit binary numbers [01] or [10] as the output. Performance was evaluated using *k*-fold (*k* = 5) cross-validation, with AUC as the performance metric. Both PCA (number of components = 30) and SVM (with the linear kernel) was implemented using Scikit-Learn (Pedregosa et al., 2011), which is a machine learning library based on Python.

### 2.6 Data and code availability

DL-EEG has great potential. Of 156 DL-EEG papers published between 2010 and 2018, Roy et al. (2019) found a median accuracy gain of 5.4% over traditional methods. However, the authors warn that most analyses would be “hard or impossible to reproduce given the unavailability of their data and code” (Roy et al., 2019, pp 1). On this point we make every effort to make our results reproducible. Scripts will be publicly available on GitHub^1^, and the EEG data will be available upon reasonable request. Lastly, we follow Roy and colleagues’ (2019) checklist of items to include in a DL-EEG study.

## 3 Results

### 3.1 Convolutional neural network

**Fig. 2:**
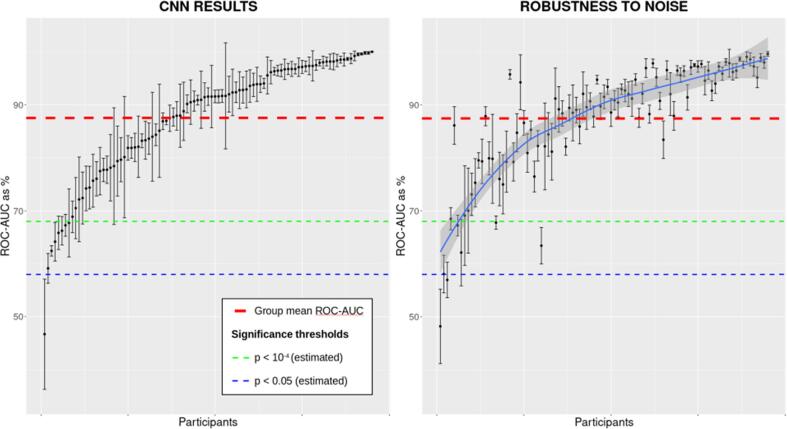
The CNN classification AUC scores of all 95 infants. Main model (left panel), robustness to noise analysis (right panel). Vertical bars represent the standard deviation of the mean for each participant and are comprised of 5 scores - the AUC score of each *k*-fold. Sequential ordering of participants is preserved across plots, and the line of best fit (right panel, blue) is included to depict the overall similarity of performance between the two datasets. The upper dashed line denotes the group mean, the middle line corresponds to p = 0.0001, and the bottom line to p = 0.05.

Based on the work of Combrisson and Jerbi (2015), accuracy scores above 58% are estimated to be significant to *p* < 0.05, and scores above 68% significant to *p* < 10^-4^, for a binary classifier of n = 100. Our first analysis investigated whether the CNN could classify which rhythmic stimulus was presented to the infant. Based on data from 95 infants, we achieved a mean AUC of 0.874 (SD = 0.11), well above the upper significance threshold (see Fig. 2 – left panel).

Our second analysis tested whether the CNN was robust against two different levels of noise. To achieve this, we ran the CNN with all data (i.e., including the “noisy” epochs rejected during preprocessing). Although there were differences on an individual level, visible in Fig. 2, overall model performance was barely affected (AUC = 0.875, SD = 0.11; Fig. 2 – right panel).

**Fig. 3:**
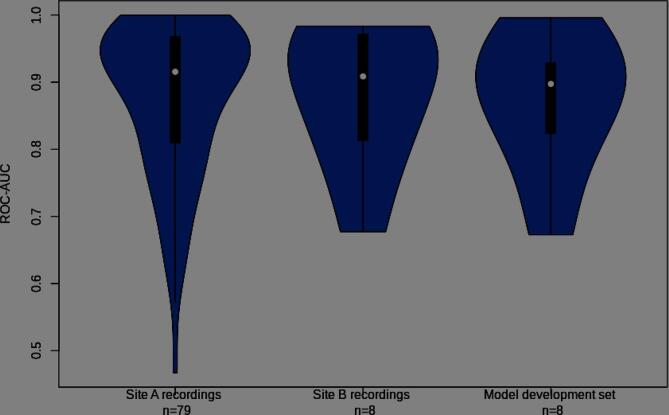
Violin plots showing the distribution of AUC scores for the CNN by recording site, and set.

Finally, the generalization capability of the CNN was assessed by comparing differences in classification performance between data recorded with different instruments. We found no significant difference in AUC scores between data recorded at site A vs. site B (Wilcoxon unpaired signed-rank test: W = 317, Z = 0.00, p = .99). In addition, there was no significant difference between the model development subset, and the data recorded at site A (Wilcoxon unpaired signed-rank test: W = 340, Z = 0.04, p = .73); see Fig. 3.

### 3.2 Support Vector Machine

**Fig. 4:**
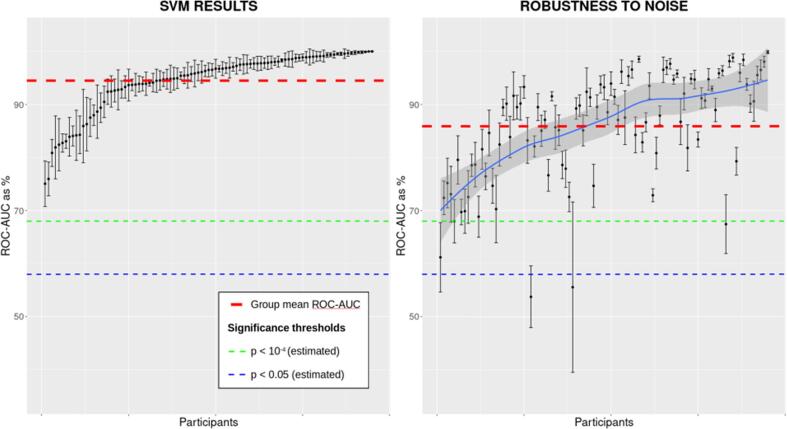
As Fig. 2, but for SVM.

The performance of the CNN was then benchmarked against that of the SVM. The average AUC score across all 95 infants using the SVM was 0.95 (SD = 0.06) for the cleaned data. However, for the data that included the “noisy” epochs rejected during preprocessing, the AUC fell to 0.86 (SD = 0.1). The SVM classification AUC scores for each level of noise are shown in Fig. 4, for comparison with Fig. 2.

## 4 Discussion

Using two machine learning approaches, we show here that non-invasive scalp EEG recordings from 8-week-old infants can be used to determine which natural rhythmic stimulus (speech vs. drumbeat) was heard with over 0.86 mean AUC even when “noisy” EEG epochs (those typically rejected during the pre-processing of infant data) were retained. For the CNN approach, we demonstrate that the modelling is sufficiently robust to cope with variability in the noisiness of the input, as classification was at 0.87 mean AUC for both the cleaned and noisy EEG datasets. An SVM (more traditional machine learning method) approach surpassed the CNN when given the cleaner dataset (mean AUC of 0.95), however it was less robust against the nosier dataset (mean AUC of 0.86). In particular, participants at the upper end of the ROC-AUC-as-% curve in Fig. 4 showed considerable changes. Hence although the SVM performed better than the CNN model with the smaller (cleaner) dataset, the CNN was more robust to the noise that is inherent in infant EEG recordings. This suggests that for infant EEG data, a CNN approach may be preferable. Accordingly, our modelling provides a proof-of-concept demonstration that rhythmic acoustic stimuli presented to infants of this age generate differential brain responses, responses which could provide eventual biomarkers of individual differences in language development. We are currently tracking the language skills of the participating infants longitudinally, in order to investigate whether individual differences in the neural response to syllables, drumbeats or both best predicts later language acquisition. Meanwhile, our data highlight the potential for an EEG-based early screening tool for language disorder that involves listening to simple rhythmic stimuli.

Traditional EEG artefact detection techniques use statistics to search for noise (e.g., independent component analysis, discrete wavelet transformation), however there is no consensus regarding best methods (Urigüen & Garcia-Zapirain, 2015), particularly for infant data (Fujioka et al., 2011, Noreika et al., 2020). A CNN can instead search for features while ignoring noise, thereby keeping raw data intact. Here we found that removing noisy EEG epochs (which accounted for approximately 25% of the data) did not change model performance for the CNN at the group level. In addition, we refrained from using artefact correction/rejection measures, in a bid to keep the data as intact as possible, although we did interpolate bad channels. Our data therefore partially support the notion proposed by Craik et al., 2019, Roy et al., 2019 that some traditional pre-processing steps may not be required for machine learning approaches to EEG analysis, given the CNN’s ability to cut through noise. Future research could systematically strip away pre-processing steps and quantify the effects on network performance. Note however that the CNN developed here was trained and tested on data from each individual infant - a within-subjects design. Accordingly, further work testing whether a network trained on one set of infants could generalise to different infants is now required. Further, we note that this type of modelling can sacrifice interpretability for performance (i.e. the “black box problem” of deep learning; Guidotti et al., 2018).

In conclusion, we have demonstrated that both CNN and SVM algorithms designed to find neural patterns *within* participants, relevant to identifying individual differences in automatic neural responses to rhythmic inputs, can achieve a high level of classification accuracy. A CNN or an SVM trained on one set of infants could in principle generalise to new infants; this remains to be demonstrated. Nevertheless, the current modelling represents an important first step in this regard, showing that infant brains are indeed responding differentially to speech vs non-speech stimuli presented at the same rhythmic rate.

## Declaration of Competing Interest

The authors declare that they have no known competing financial interests or personal relationships that could have appeared to influence the work reported in this paper.

## Footnotes

1 https://github.com/samuel-gibbon/BabyRhythm
